# The Multi‐Functional Effects of CuS as Modifier to Fabricate Efficient Interlayer for Li‐S Batteries

**DOI:** 10.1002/advs.202204561

**Published:** 2022-10-26

**Authors:** Mengzi Geng, Hangqi Yang, Chaoqun Shang

**Affiliations:** ^1^ School of Material Science and Engineering & Hubei Key Laboratory of Plasma Chemistry and Advanced Materials Wuhan Institute of Technology Wuhan 430205 China; ^2^ School of Resource and Environmental Sciences Wuhan University Wuhan 430072 China

**Keywords:** CuS/CNTs interlayer, high S loading, lean electrolyte, Li–S batteries, shuttle effect

## Abstract

The shuttle effect of lithium polysulfides in lithium‐sulfur batteries (LSBs) has a detrimental impact on their electrochemical performance. To effectively mitigate the shuttle effect, in this study, the coral‐like CuS is introduced to modify the carbon nanotube (CNTs), which is coated on commercial separator and served as the S cathode interlayer (PE@CuS/CNTs). The CuS/CNTs interlayer possesses efficient physical impediment and chemisorption to polysulfide anions. When achieving maximum adsorption to polysulfide anions, a “polysulfide‐phobic” surface would be formed as a shield to restrain the polysulfide anions in the cathode region. Simultaneously, the CuS/CNTs interlayer can improve the lithium ion diffusion and guarantee desirable electrochemical reaction kinetics. Consequently, the LSBs with PE@CuS/CNTs show an initial discharge capacity of 1242.4 mAh g^−1^ at 0.5 C (1 C = 1675 mA g^−1^) and retain a long‐term cycling stability (568.5 mAh g^−1^ after 1000 cycles, 2 C), corresponding to an ultra‐low capacity fading rate of only 0.05% per cycle. Also, the LSBs with PE@CuS/CNTs exhibit high resistance to self‐discharge and favorable performance under high S loading (4.5 mg cm^−2^) and lean electrolyte (9.4 mL_Electrolyte_ g _S_^−1^).

## Graphical abstract

The CuS/CNTs interlayer possesses efficient physical impediment and chemisorption to polysulfide anions. When achieving maximum adsorption to polysulfide anions, a “polysulfide‐phobic” surface would be formed as a shield to restrain the polysulfide anions in the cathode region. Simultaneously, the CuS/CNTs interlayer can improve the Li^+^ and guarantee desirable electrochemical reaction kinetics.

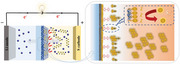

## 1 Introduction

The rapid development of new‐energy vehicles and mobile electrical equipment needs further investigation on power batteries with longer service lives and higher energy density. Great research interests have been attracted by lithium‐sulfur batteries (LSBs) owing to their appealing theoretical energy density of 2600 Wh kg^−1^.^[^ ^1^, ^2^, ^3^, ^4^, ^5^, ^6^ ^]^ However, the commercial development of LSBs is impeded by many challenges, such as low S utilization, unstable Li deposition, and undesirable electrochemical performance. Most importantly, in the cathode region, the low conductivity of S and Li_2_S_2_/Li_2_S, combined with the notorious shuttle effect of soluble lithium polysulfides (LiPSs), leads to severe self‐discharge and poor cycle performance.^[^ ^7^, ^8^, ^9^, ^10^, ^11^, ^12^, ^13^ ^]^

Many efforts have been made to conquer the above‐mentioned hinderances, especially for addressing the shuttle effect of LiPSs. In the early stage, various carbon materials, such as carbon nanotubes (CNTs),^[^ ^14^, ^15^ ^]^ mesoporous carbon^[^ ^16^, ^17^ ^]^ and graphene nanosheets^[^ ^18^ ^]^ are composited with S to confine the S species in their spaces through intermolecular interactions or capillary forces. However, due to the intrinsic nonpolar nature, the structure of carbon‐based host materials will gradually collapse with the accumulation of LiPSs, especially in the case of high S loading.^[^ ^19^, ^20^, ^21^, ^22^, ^23^, ^24^ ^]^ Additionally, the excessive use of host materials increases the weight of positive electrode, reducing the S mass ratio and total energy density of LSBs.

An effective tactic is to insert a polysulfide barrier between the separator and the S cathode without compensate the S content in the cathode.^[^ ^25^, ^26^, ^27^ ^]^ The interlayer with relatively low content should be selectively permeable, allowing viable Li^+^ transport but preventing the diffusion of polysulfide anions. Usually, the carbon materials with high conductivity are employed as the interlayer owing to their low cost, light weight, chemical stability, and feasible manufacture.^[^ ^28^ ^]^ Unfortunately, the nonpolar carbon interlayer shows limited adsorption capability to polar LiPSs, which always acts as physical barrier and upper current collector to restrain the S species in the cathode side with insufficient confinement during repeated cycles. Conversely, metal sulfides, as polar materials, can form strong chemical interactions with LiPSs,^[^ ^29^, ^30^, ^31^, ^32^, ^33^, ^34^, ^35^, ^36^, ^37^ ^]^ while their low conductivity increases the internal resistance,^[^ ^38^, ^39^, ^40^, ^41^ ^]^ impeding the S utilization. Thus, the rational design based on the synergistic combination of nonpolar carbon materials and polar metal sulfides as interlayer provides a feasible way to effectively suppress the shuttle effect of LiPSs in LSBs during cycling.^[^ ^42^, ^43^, ^44^, ^45^, ^46^ ^]^ Nevertheless, in consideration of the low content of interlayer in LSBs, the adsorption of LiPSs is limited especially at high S loading, which is always neglected in most cases.^[^ ^47^ ^]^

The polar CuS, inexpensive and readily available, is considered to trap LiPSs by strongly chemical interaction.^[^ ^48^, ^49^, ^50^ ^]^ Besides, the CuS has a smaller Li^+^ diffusion barrier (0.15 eV) according to previous reports, which is beneficial to the Li^+^ diffusion.^[^ ^48^, ^51^, ^52^ ^]^ In this work, in combination with highly electrical conductive CNTs providing long‐range electron pathway, the CuS/CNTs are fabricated and coated on commercial polyethylene separator (denoted as PE@CuS/CNTs). As a functional interlayer for LSBs, the CuS/CNTs have several promises: (1) forming upper current collector to guarantee sufficient electron transfer, (2) providing physical barrier to hinder the transport of LiPSs, (3) adsorbing LiPSs via the strong chemical bonding of CuS to polysulfides, (4) constructing a “polysulfide‐phobic” surface after polar CuS effectively adsorbing a certain amount of polysulfides and providing repulsive force to the unreacted polysulfides. Benefited from these issues, the soluble LiPSs can be effectively confined in the cathode region during the LSB operation. As a consequence, the LSBs with PE@CuS/CNTs show excellent electrochemical performances, including a high first‐lap capacity (1242.4 mAh g^−1^, 0.5 C), strong self‐discharge suppression capability (87% capacity retention after 120 h of rest) and excellent long‐term stability (568.5 mAh g^−1^ after 1000 cycles, 2 C).

## 2 Results and Discussion

**Figure 1:**
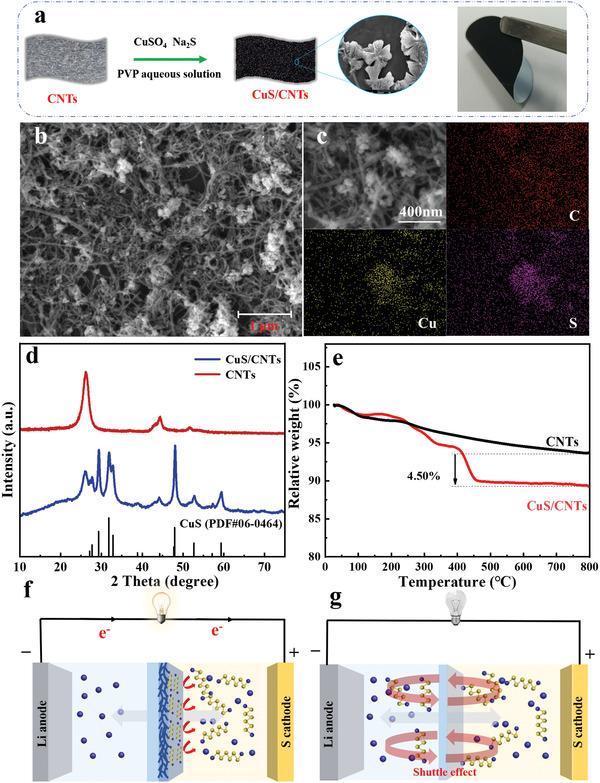
a) Schematic preparation process of PE@CuS/CNTs. b) SEM and c) SEM and corresponding EDS mapping images of CuS/CNTs. d) XRD and e) TGA results of CuS/CNTs and CNTs. The schemes of LSBs equipped f) with and g) without CuS/CNTs interlayer.

**Figure** **1**a illustrates the fabrication of CuS/CNTs, where the CuS is formed by the reaction between Na_2_S and CuSO_4_ with the aid of polyvinyl‐pyrrolidone in aqueous solution at room temperature. The preparation process of CuS is relatively easy and simple without high temperature treatment or even hydrothermal procedure. The commercial CNTs with large length–diameter ratio (Figure S1, Supporting Information) can serve as the substrate for the formation of coral‐like CuS (Figure S2, Supporting Information). The CuS/CNTs appear as a tangled and interwoven CNT network with the CuS flakes evenly distributed inside the CNT‐network as verified by the field‐emission scanning electron microscope (SEM, Figure 1b). The SEM and corresponding energy dispersive spectroscopy (EDS) mapping images of CuS/CNTs in Figure 1c further demonstrate the dispersive growth of CuS on CNTs network. Figure 1d depicts the X‐ray diffraction (XRD) pattern of CuS/CNTs, where the characteristic diffraction peaks at 27.7°, 29.3°, 31.8°, 32.9°, 38.8°, 47.9°, 52.7°, and 59.3° are corresponding to the (101), (102), (103), (006), (105), (110), (108), and (116) planes of CuS (PDF#06‐0464), respectively. Additionally, the peaks appearing at 25° and 44.8° are consistent with the characteristic peaks of carbon in CNTs. As shown in Figure 1e of thermogravimetric analysis (TGA), the mass loss of CuS/CNTs below 120 °C is mainly caused by the evaporation of absorbed water molecules. With increasing the temperature from ≈155 to ≈515 °C, the CuS in CuS/CNTs is transformed to Cu_2_S with continuous S evaporation, during which the formed polymeric phase including digenite, Cu_1.8_S and chalcocite is responsible to the complicated multiple mass loss steps.^[^ ^53^ ^]^ According to the law of conservation of mass,^[^ ^39^, ^48^ ^]^ the content of CuS in CuS/CNTs is 24.97%. According to the N_2_ adsorption–desorption curves in Figure S3 (Supporting Information), the CuS/CNTs has BET surface area of 62.73 m^2^ g^−1^ with mesoporous pores, which can facilitate the immobilization of LiPSs, further suppressing the LiPSs’ shuttle effect. As further verified by adding CuS/CNTs into the Li_2_S_6_ solution, the corresponding solution turns into lighter color with lower UV–visible spectra intensity in the characteristic Li_2_S_6_ region of 400–500 nm than that of CNTs after 4 h (Figure S5, Supporting Information).

The as‐obtained CuS/CNTs are coated on commercial PE separator with the assistance of poly(vinylidene difluoride) (More experimental details in the Supporting Information). The thickness of CuS/CNTs interlayer on PE is about 22 µm (Figure S4, Supporting Information) with low mass loading of ≈0.3 mg cm^−2^. And the corresponding PE@CuS/CNTs shows high flexibility (inset of Figure 1a), which might improve the tolerance under large volume variation during repeated charge–discharge processes in LSBs. The schematic diagram of LSBs using PE@CuS/CNTs is shown in Figure 1f, where the CuS/CNTs’ physical hindrance and chemical adsorption can keep polysulfides well confined in the cathode region but Li^+^ can move freely and guarantee stable redox reaction progress. However, when applying traditional PE without CuS/CNTs, a severe shuttle effect is unavoidable, resulting in undesirable electrochemical performance (Figure 1g).

**Figure 2:**
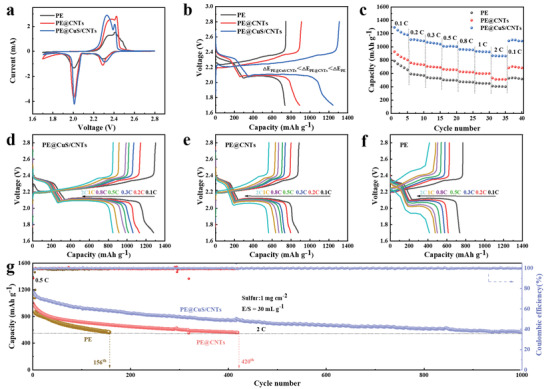
a) CV curves at 0.1 mV s^−1^, b) GCD curves at 0.5 C, c) rate capability, d–f) corresponding GCD curves at various rates, and g) cycling performance at 2 C of LSBs with PE@CuS/CNTs, PE@CNTs, and PE.

The electrochemical performance of LSBs’ interlayers with different CuS/CNTs ratios at 0.5 C was first tested to optimize the raw‐material ratio of CuS to CNTs (Figure S6, Supporting Information). When the ratio of CuS to CNTs is 1:2, the LSBs show the best performance where a discharge capacity of 837.8 mAh g^−1^ is achieved (after 100 cycles, 0.5 C). Therefore, the CuS/CNTs (1:2) interlayer is employed for further investigation in the following tests. As shown in **Figure** **2**a, two distinct reduction peaks are shown in the cyclic voltammetry (CV) curves, corresponding to the conversion of cyclo‐S_8_ to long‐chain Li_2_S_4–8_ and further reaction to Li_2_S_2_ or Li_2_S. The LSBs with PE@CuS/CNTs show higher peak currents and lower overpotentials between the oxidation and reduction peaks, revealing increased S utilization and enhanced reaction kinetics. The initial galvanostatic‐charge/discharge (GCD) curves of LSBs with PE@CuS/CNTs at 0.5 C show flatter plateaus and lower overpotential than those of PE and PE@CNTs (Figure 2b). Obviously, the lower overpotential is mainly derived from the improved charge process. This phenomenon is might be due to the partial Cu formation during discharge (Figures S7 and S8, Supporting Information). Although the low CuS loading has a negligible effect on capacity, the in situ generated Cu can effectively improve the conductivity of the CuS/CNTs network, which is beneficial in lowering the charge overpotential in the subsequent charge process. Therefore, the highest initial capacity (1242.4 mAh g^−1^ at 0.5 C) is obtained for the LSBs with PE@CuS/CNTs. Additionally, after 200 cycles, the discharge capacity of the LSBs with PE@CuS/CNTs (834.2 mAh g^−1^) is higher than those of the LSBs using PE@CNTs (566.6 mAh g^−1^) and PE (353 mAh g^−1^) (Figure S9, Supporting Information). As shown in the ex situ top‐view SEM images of PE@CuS/CNTs and PE@CNTs after 200 cycles (Figure S10, Supporting Information), CuS/CNTs interlayer shows lower signal of S species than that of CNTs interlayer. This is might be attributed to the “polysulfide‐phobic” surface, which would repel and confine the LiPSs anions in cathode region.

As for the rate capability depicted in Figure 2c,d, the LSBs with PE@CuS/CNTs deliver high discharge capacities of 1292.5, 1110.1, 1070.1, 1009.8, 963.3, 933.0, and 867.6 mAh g^−1^ at 0.1, 0.2, 0.3, 0.5, 0.8, 1, and 2 C, respectively. More importantly, when the current density returns to 0.1 C, the discharge capacity (1087.6 mAh g^−1^) can be recovered and remain stable, showing excellent reversible capacity retention after a series of high‐rate tests. Without CuS, the discharge capacities of LSBs using PE@CNTs (Figure 2e) and PE (Figure 2f) are 510.1 and 400.5 mAh g^−1^ as the current density increased to 2 C, respectively. The long‐term cycling stability of LSBs using PE@CuS/CNTs at 2 C was also investigated. The LSBs with PE@CuS/CNTs show an initial capacity of 1150 mAh g^−1^, which retains of 568 mAh g^−1^ after 1000 cycles, corresponding to an ultralow capacity fading rate of 0.05% per cycle as shown in Figure 2g. Conversely, the LSBs with PE or PE@CNTs show rapid capacity fading and lower capacity retention.

**Figure 3:**
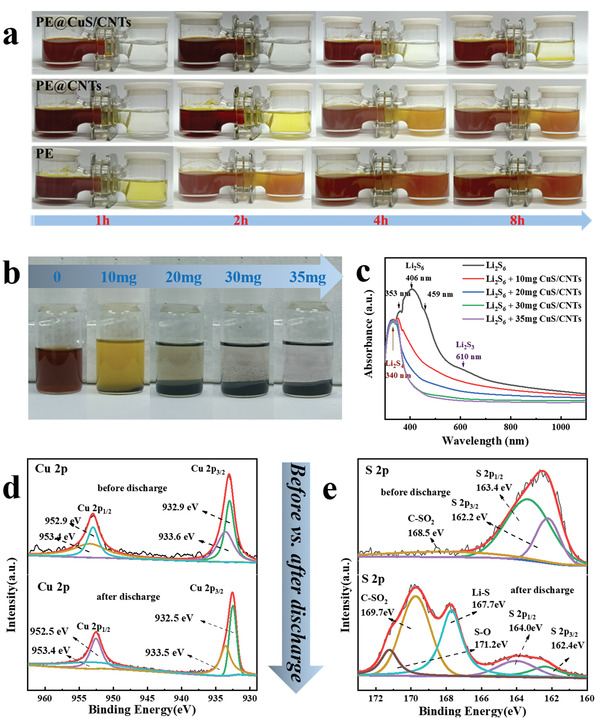
a) Li_2_S_6_ (50 mm) permeation tests of PE@CuS/CNTs, PE@CNTs, and PE. b) Visual adsorption and c) corresponding UV–vis results of 5 mL Li_2_S_6_ (10 mm) solution upon adding different amounts of CuS/CNTs. High‐resolution XPS spectra of d) Cu 2p and e) S 2p of CuS/CNTs interlayer before and after discharge.

As discussed above, the LSBs with PE@CuS/CNTs deliver considerable electrochemical performance. To gain more insights into the effect of CuS, the physicochemical barrier and adsorption effects of various separators to Li_2_S_6_ are compared by the permeability test as an example of LiPSs with H‐type cells (**Figure** **3**a). After 1 h, the solution on the right changes to yellow in the equipment with PE separator. Moreover, the colors on both sides are nearly identical after 4 h, expressing that the PE is not able to restrain Li_2_S_6_. Meanwhile, the right‐side solution of the PE@CNT separator is lighter than that of the PE, indicating that CNTs have a certain physical blocking effect on LiPSs. But there is still a lot of Li_2_S_6_ infiltrating the right. Surprisingly, only a faint color change is shown in the solution with the CuS/CNTs interlayer after standing for 8 h, demonstrating that the synergistic of chemical adsorption of CuS and physical confinement of CNTs can effectively prevent the diffusion of Li_2_S_6_. To estimate the maximum adsorption of CuS/CNTs to Li_2_S_6_, different amounts of CuS/CNTs were added into Li_2_S_6_. As shown in Figure 3b, 30 mg CuS/CNTs can effectively change the Li_2_S_6_ solution from dark orange to colorless, which is further verified by corresponding UV–vis spectra (Figure 3c). We can speculate that the adsorption of CuS/CNTs to Li_2_S_6_ is ≈1.67 × 10^−3^ mmol (Li_2_S_6_)/mg (CuS/CNTs). During battery operation in realistic system, the mass loading of CuS/CNTs is ≈0.3 mg cm^−2^ (*Φ* = 1.9 cm), corresponding to 1.42 × 10^−3^ mmol Li_2_S_6_ maximum adsorption (more calculation details in the Supporting Information). Only with 1 mg cm^−2^ S loading, the ideally transformed Li_2_S_6_ is ≈5.9 × 10^−3^ mmol during the discharge process, which is more than three times to that of maximum adsorption, let alone in the case of 4.5 mg cm^−2^. Thus, a reasonable speculation is that the CuS/CNTs with fully adsorbed LiPSs will be relatively negatively charged and self‐construct a “polysulfide‐phobic” surface, generating a repulsive force with the negative polysulfide ions and further blocking LiPSs in the cathode region.

The XPS tests were performed on the CuS/CNTs interlayer before and after discharge to get a deep comprehension of the chemical interaction between CuS/CNTs and S species. As shown in Figure 3d of the high‐resolution XPS of Cu 2p, the fitted peaks of Cu 2p_3/2_ are predominantly concentrated at 932.9 and 933.6 eV, while Cu 2p_1/2_ has two deconvoluted peaks at 952.9 and 953.4 eV. After discharge, the peaks of both Cu 2p_3/2_ and Cu 2p_1/2_ slightly shift to lower binding energy. This may be ascribed to the electrons transferred from negatively charged polysulfide anions to positive Cu^2+^ through the strong chemical affinity.^[^ ^48^, ^49^, ^54^ ^]^ The peaks at 162.2 and 163.4 eV in Figure 3e are ascribed to S 2p_3/2_ and S 2p_1/2_, which positively shift to 162.4 and 164.0 eV after discharge, suggesting that the electrons are transferred from the surface of S atoms to Li atoms. Additionally, the new peak at 167.7 eV is ascribed to the generation of Li_2_S. Furthermore, the peak at 171.2 eV is due to the S—O bond in the reaction products.^[^ ^5^, ^51^, ^55^ ^]^ The above results indicate that there is a strong chemisorption between polysulfides and CuS, which accelerates the immobilization of S species during the electrochemical process. These features are responsible to the LSBs’ superior capacity and cycle stability.

**Figure 4:**
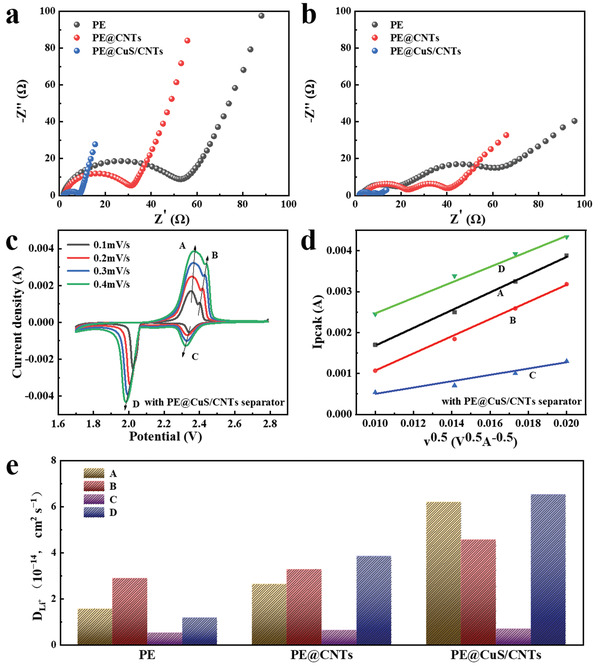
EIS of the LSBs with PE@CuS/CNTs, PE@CNTs, and PE a) before and b) after 200 cycles at 0.5 C. c) CV curves at different scan rates and d) corresponding linear fits (peak current versus *v* ^0.5^) of LSBs with PE@CuS/CNTs. e) *D*_Li+_ summarization of LSBs with PE@CuS/CNTs, PE@CNTs, and PE.

An ideal cathode interlayer of LSBs should not only restrain polysulfides but also improve the Li^+^ transport. As shown in **Figure** **4**a, the electrochemical impedance spectroscopy (EIS) profiles of the fresh LSBs with different separators consist of a semicircle and a low frequency tail, corresponding to the charge‐transfer resistance and Warburg impedance, respectively. As predicted, the LSBs with PE@CuS/CNTs delivers smaller charge‐transfer resistance (*R*_ct_), implying faster transport of Li^+^ in the interlayer due to the highly conductive and cross‐stacked CuS/CNTs network. Another semicircle appeared in the mid‐frequency region after 200 cycles (Figure 4b). This is due to the charge‐transfer resistance of LiPSs that migrate to non‐chemically active sites during the long‐term cycling process. Additionally, the smaller mid‐frequency semicircle shows that CuS/CNTs effectively limit the migration of polysulfide anions during repeated cycles.

To further explore the influence of CuS/CNTs on Li^+^ diffusion, the Li^+^ diffusion coefficient (*D*_Li+_) is calculated by the Randles–Sevick equation (more information in the Supporting Information) using CV peak currents at various scan rates (Figure 4c).^[^ ^56^, ^57^ ^]^ As shown in Figure 4d and Figure S11 (Supporting Information), the *I*_p_ (peak current) and *v* ^0.5^ (*v* is the scan rate) show a good linear relationship, demonstrating that the electrochemical reactions of LSBs are controlled by ion diffusion. Because the *D*_Li+_ value is proportional to *I*_p_/*v* ^0.5^, the slope of the obtained straight line can be used to determine the magnitude of *D*_Li+_. As summarized in Figure 4e, the *D*_Li+_ of each peak in PE@CuS/CNTs is higher than those of PE@CNTs and PE, which further proves that the CuS/CNTs interlayer is beneficial to the Li^+^ migration.

**Figure 5:**
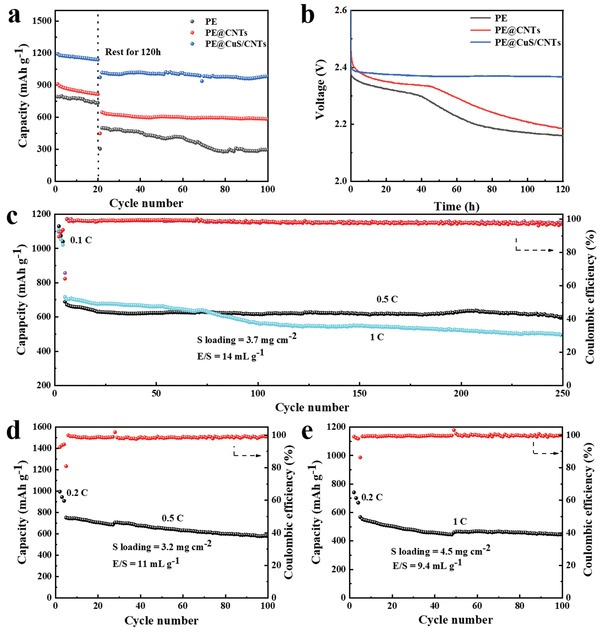
a) Cycling performances of LSBs with PE@CuS/CNTs, PE@CNTs, and PE before and after interruption at 0.5 C and b) corresponding voltage curves during interruption. Cycling performance of LSBs with PE@CuS/CNTs: c) 0.5 and 1 C, S loading = 3.7 mg cm^−2^, E/S = 14 mL g^−1^, d) 0.5 C, S loading = 3.2 mg cm^−2^, E/S = 11 mL g^−1^, and e) 1 C, S loading = 4.5 mg cm^−2^, E/S = 9.4 mL g^−1^.

Generally, the LiPSs derived from the dissolution of active S will diffuse to the Li anode resulting in the occurrence of significant self‐discharge during a prolonged break. Here, the introduction of CuS/CNTs also contributes to improved anti‐self‐discharge capability of LSBs. As clarified in **Figure** **5**a, following the initial 20 cycles at 0.5 C, the capacity retention after 120 h interruption is 85.8% and the discharge capacity remained at nearly 950 mAh g^−1^ after 100 cycles for LSBs with PE@CuS/CNTs. Under the same condition, the LSBs with PE@CNTs or pristine PE exhibit much lower capacity retention of only 60.1% or 51.2%, respectively. The open‐circuit voltages during quiescence of LSBs with different separators are shown in Figure 5b. Obviously, the LSBs using PE@CuS/CNTs maintained a relatively stable open‐circuit voltage, staying around at 2.36 V during the 120 h resting period, which is superior to those of PE@CNTs (2.19 V) and PE (2.16 V) with continuous voltage decline. These results demonstrate that the shuttle effect of LiPSs is effectively alleviated under the double effects of physical interception and chemisorption conversion of CuS/CNTs, thereby significantly suppressing the self‐discharge.

To meet the practical requirement, the LSBs with PE@CuS/CNTs were assembled under the condition of S loading of 3.7 mg cm^−2^ and the electrolyte content/S loading (E/S) ratio of 14 mL g^−1^. The LSBs displayed an acceptable capacity of 689.4 mAh g^−1^ at 0.5 C after activation for 3 cycles (Figure 5c). After 250 cycles, the capacity remained at 603.1 mAh g^−1^, corresponding to a capacity retention rate of 87.5%. In addition, at 1 C, the LSBs with PE@CuS/CNTs still deliver a capacity of 500 mAh g^−1^. The lower E/S ratio may contribute to the improvement of LSBs’ overall energy density. Decreasing the E/S ratio to 11 mL g^−1^ (Figure 5d), the LSBs show an initial capacity of 751.6 mAh g^−1^ and retained a capacity of 579.3 mAh g^−1^ after 100 cycles at 0.5 C with a S loading of 3.2 mg cm^−2^. After continuously reducing the E/S ratio to 9.4 mL g^−1^ and increasing the S loading to 4.5 mg cm^−2^, the LSBs retain a capacity of 446 mAh g^−1^ at 1 C (Figure 5e). The Coulombic efficiency of LSBs always remains above 98% within 100 cycles, and the capacity retention is >77%. Compared with reported results with similar approach, the CuS/CNTs interlayer shows comparable electrochemical property, especially in long‐cycle and high‐loading performances (Table S1, Supporting Information).^[^ ^12^, ^16^, ^22^, ^23^, ^24^, ^31^ ^]^

## 3 Conclusions

A CuS/CNTs interlayer has been introduced to inhibit the LSBs’ shuttle effect. The synergy effect between the CNTs network with high conductivity and the strong physicochemical adsorption of CuS to LiPSs promises suppressed shuttle effect and enhanced reaction kinetics. Therefore, the LSBs with PE@CuS/CNTs exhibit considerable performances in various electrochemical properties, including long‐term cycling stability (568.5 mAh g^−1^ after 1000 cycles), high‐rate capability, and self‐discharge resistance (87% capacity retention after 120 h of rest). Even after 100 cycles with high S loading (4.5 mg cm^−2^) and lean electrolyte (9.4 mL g^−1^), the corresponding LSBs maintain a high specific capacity of 446 mAh g^−1^.

## Conflict of Interest

The authors declare no conflict of interest.

## Supporting information

Supporting Information

## Data Availability

The data that support the findings of this study are available from the corresponding author upon reasonable request.

## References

[1] A. Manthiram , Y. Fu , S. Chung , C. Zu , Y. Su , Chem. Rev. 2014, 114, 11751. 25026475 10.1021/cr500062v

[2] Q. Pang , X. Liang , C. Kwok , L. Nazar , Nat. Energy 2016, 1, 132.

[3] X. Ji , K. T. Lee , L. F. Nazar , Nat. Mater. 2009, 8, 500. 19448613 10.1038/nmat2460

[4] Z. Seh , Y. Sun , Q. Zhang , Y. Cui , Chem. Soc. Rev. 2016, 45, 5605. 27460222 10.1039/c5cs00410a

[5] J. Chen , H. Zhang , H. Yang , J. Lei , A. Naveed , J. Yang , Y. Nuli , J. Wang , Energy Storage Mater. 2020, 27, 307.

[6] X. Wang , Y. Zhao , F. Wu , S. Liu , Z. Zhang , Z. Tan , X. Du , J. Li , J. Energy Chem. 2021, 57, 19.

[7] X. Yuan , X. Zhao , J. Hu , Z. Li , Y. Qin , Y. Peng , Z. Deng , Chem. Eng. J. 2021, 426, 131355.

[8] X. Li , A. Lushington , Q. Sun , W. Xiao , J. Liu , B. Wang , Y. Ye , K. Nie , Y. Hu , Q. Xiao , R. Li , J. Guo , T. K. Sham , X. Sun , Nano Lett. 2016, 16, 3545. 27175936 10.1021/acs.nanolett.6b00577

[9] X. Jiang , S. Zhang , B. Zou , G. Li , S. Yang , Y. Zhao , J. Lian , H. Li , H. Ji , Chem. Eng. J. 2022, 430, 131911.

[10] H. Shao , W. Wang , H. Zhang , A. Wang , X. Chen , Y. Huang , J. Power Sources 2018, 378, 537.

[11] Z. Fan , C. Zhang , W. Hua , H. Li , Y. Jiao , J. Xia , C. Geng , R. Meng , Y. Liu , Q. Tang , Z. Lu , T. Shang , G. Ling , Q. Yang , J. Energy Chem. 2021, 62, 590.

[12] G. Wang , Y. Lai , Z. Zhang , J. Li , Z. Zhang , J. Mater. Chem. A 2015, 3, 7139.

[13] X. Huang , B. Luo , P. Chen , D. J. Searles , D. Wang , L. Wang , Coord. Chem. Rev. 2020, 422, 213445.

[14] Z. Sun , T. Wang , Y. Zhang , K. Kempa , X. Wang , Electrochim. Acta 2019, 327, 134843.

[15] S. Li , H. Zhang , W. Chen , Y. Zou , H. Yang , J. Yang , C. Peng , ACS Appl. Mater. Interfaces 2020, 12, 25767. 32406669 10.1021/acsami.0c03182

[16] J. Zhang , C. Yang , Y. Yin , L. Wan , Y. Guo , Adv. Mater. 2016, 28, 9539. 27620697 10.1002/adma.201602913

[17] G. Ma , Z. Wen , J. Jin , M. Wu , X. Wu , J. Zhang , J. Power Sources 2014, 267, 542.

[18] D. Liu , C. Zhang , G. Zhou , W. Lv , G. Ling , L. Zhi , Q. H. Yang , Adv. Sci. 2018, 5, 270. 10.1002/advs.201700270 PMC5770674 29375960

[19] X. Rui , H. Tan , Q. Yan , Nanoscale 2014, 6, 9889. 25073046 10.1039/c4nr03057e

[20] Y. Jeong , J. Kim , S. Nam , C. Park , S. Yang , Adv. Funct. Mater. 2018, 28, 7411.

[21] R. Fang , S. Zhao , S. Pei , X. Qian , P. X. Hou , H. M. Cheng , C. Liu , F. Li , ACS Nano 2016, 10, 8676. 27537348 10.1021/acsnano.6b04019

[22] S. Bai , X. Liu , K. Zhu , S. Wu , H. Zhou , Nat. Energy 2016, 1, 96.

[23] F. Hu , H. Peng , T. Zhang , W. Shao , S. Liu , J. Wang , C. Wang , X. Jian , J. Energy Chem. 2021, 58, 115.

[24] L. Meng , Y. Li , Q. Lin , J. Long , Y. Wang , J. Hu , ACS Appl. Energy Mater. 2021, 4, 8592.

[25] X. Wang , X. Zhao , C. Ma , Z. Yang , G. Chen , L. Wang , H. Yue , D. Zhang , Z. Sun , J. Mater. Chem. A 2020, 8, 1212.

[26] L. Fan , M. Li , X. Li , W. Xiao , Z. Chen , J. Lu , Joule 2019, 3, 361.

[27] B. Guan , Y. Zhang , L. Fan , X. Wu , M. Wang , Y. Qiu , N. Zhang , K. Sun , ACS Nano 2019, 13, 6742. 31184129 10.1021/acsnano.9b01329

[28] W. Kong , L. Yan , Y. Luo , D. Wang , K. Jiang , Q. Li , S. Fan , J. Wang , Adv. Funct. Mater. 2017, 27, 6663.

[29] J. He , Y. Chen , A. Manthiram , Energy Environ. Sci. 2018, 11, 2560.

[30] H. Lin , L. Yang , X. Jiang , G. Li , T. Zhang , Q. Yao , G. Zheng , J. Y. Lee , Energy Environ. Sci. 2017, 10, 1476.

[31] N. Hu , X. Lv , Y. Dai , L. Fan , D. Xiong , X. Li , ACS Appl. Mater. Interfaces 2018, 10, 18665. 29771116 10.1021/acsami.8b03255

[32] T. Deng , W. Sun , Y. Mao , J. Huang , L. He , X. Dou , Y. Bai , Z. Wang , K. Sun , ChemElectroChem 2022, 9, 1519.

[33] L. L. Kong , Z. Zhang , Y. Z. Zhang , S. Liu , G. R. Li , X. P. Gao , ACS Appl. Mater. Interfaces 2016, 8, 31684. 27805807 10.1021/acsami.6b11188

[34] Y. Jeong , J. Kim , S. Nam , C. Park , S. Yang , Adv. Funct. Mater. 2018, 28, 1707411.

[35] E. H. M. Salhabi , J. Zhao , J. Wang , M. Yang , B. Wang , D. Wang , Angew. Chem., Int. Ed. Engl. 2019, 58, 9078. 31115155 10.1002/anie.201903295

[36] K. Tan , Y. Liu , Z. Tan , J. Zhang , L. Hou , C. Yuan , J. Mater. Chem. A 2020, 8, 3048.

[37] J. Wang , Y. Cui , D. Wang , Nanoscale Horiz. 2020, 5, 1287. 32735007 10.1039/d0nh00311e

[38] Q. Hao , G. Cui , Y. Zhang , J. Li , Z. Zhang , Chem. Eng. J. 2020, 381, 122672.

[39] C. Ding , D. Su , W. Ma , Y. Zhao , D. Yan , J. Li , H. Jin , Appl. Surf. Sci. 2017, 403, 1.

[40] Y. Liu , Z. Zhou , S. Zhang , W. Luo , G. Zhang , Appl. Surf. Sci. 2018, 442, 711.

[41] Z. Tan , S. Liu , X. Zhang , J. Wei , Y. Liu , L. Hou , C. Yuan , J. Mater. Chem. A 2022, 10, 18679.

[42] Z. Xu , Z. Wang , M. Wang , H. Cui , Y. Liu , H. Wei , J. Li , Chem. Eng. J. 2021, 422, 130049.

[43] J. Zhang , L. Feng , Y. Jian , G. Luo , M. Wang , B. Hu , T. Liu , J. Li , Y. Yuan , N. Wang , Chem. Eng. J. 2022, 429, 132265.

[44] J. Cheng , Y. Pan , J. Zhu , Z. Li , J. Pan , Z. Ma , J. Power Sources 2014, 257, 192.

[45] J. Zhao , M. Yang , N. Yang , J. Wang , D. Wang , Chem. Res. Chin. Univ. 2020, 36, 313.

[46] Z. Pei , Y. Liu , D. Sun , Z. Zhu , G. Wang , Chem. Res. Chin. Univ. 2020, 36, 631.

[47] Y. He , Y. Qiao , Z. Chang , X. Cao , M. Jia , P. He , H. Zhou , Angew. Chem., Int. Ed. Engl. 2019, 58, 11774. 31210379 10.1002/anie.201906055

[48] H. Li , L. Sun , Y. Zhao , T. Tan , Y. Zhang , Appl. Surf. Sci. 2019, 466, 309.

[49] H. Li , Y. Wang , J. Huang , Y. Zhang , J. Zhao , Electrochim. Acta 2017, 225, 443.

[50] H. Tao , X. Yang , L. Zhang , S. Ni , J. Phys. Chem. Solids 2014, 75, 1205.

[51] X. Chen , H. Peng , R. Zhang , T. Hou , J. Huang , B. Li , Q. Zhang , ACS Energy Lett. 2017, 2, 795.

[52] J. Long , H. Zhang , J. Ren , J. Li , M. Zhu , T. Han , B. Sun , S. Zhu , H. Zhang , J. Liu , Electrochim. Acta 2020, 356, 36853.

[53] M. Baláž , E. Dutková , Z. Bujňáková , E. Tóthová , N. G. Kostova , Y. Karakirova , J. Briančin , M. Kaňuchová , J. Alloys Compd. 2018, 746, 576.

[54] Y. Ren , H. Wei , B. Yang , J. Wang , J. Ding , Electrochim. Acta 2014, 145, 193.

[55] J. Xu , W. Zhang , H. Fan , F. Cheng , D. Su , G. Wang , Nano Energy 2018, 51, 73.

[56] Z. Hu , Z. Zhu , F. Cheng , K. Zhang , J. Wang , C. Chen , J. Chen , Energy Environ. Sci. 2015, 8, 1309.

[57] Y. Yang , J. Li , X. He , J. Wang , D. Sun , J. Zhao , J. Mater. Chem. A 2016, 4, 7165.

