# Supplementary material for: The Multi‐Functional Effects of CuS as Modifier to Fabricate Efficient Interlayer for Li‐S Batteries
Source: Adv Sci (Weinh). 2022 Oct 26;9(35):2204561. doi: 10.1002/advs.202204561 (PMC9762292; doi:10.1002/advs.202204561)
Supplement: Supplementary file 1 — Supporting Information [file ADVS-9-2204561-s001.pdf]

ADVANCED SCIENCE
Open Access

Supporting Information

for *Adv. Sci.*, DOI 10.1002/advs.202204561

# The Multi-Functional Effects of CuS as Modifier to Fabricate Efficient Interlayer for Li-S Batteries

*Mengzi Geng, Hangqi Yang and Chaoqun Shang**

# Supporting information for

# The Multi-functional Effects of CuS as Modifier to Fabricate Efficient Interlayer for Li-S Batteries

Mengzi Geng[1,2#], Hangqi Yang[1,2#], Chaoqun Shang[1]*

[1]School of Material Science and Engineering & Hubei Key Laboratory of Plasma Chemistry and Advanced Materials, Wuhan Institute of Technology, Wuhan, 430205, China
[2]School of Resource and Environmental Sciences, Wuhan University, Wuhan, 430072, China

*Corresponding author: chaoqun.shang@foxmail.com

#These authors contributed equally.

## Experimental Section

### Materials

The materials used were multiwalled carbonnanotubes (98%, Aladdin, CNTs), $CuSO_4$ (98%, Aladdin), sublimed S (99%, Aladdin), polyvinyl pyrrolidone (PVP, Mw=1 300 000, 98%, Aladdin), $Li_2S$ (99%, Aladdin), $Na_2S$ (98%, Aladdin), poly (vinylidene difluoride) (PVDF 1015, Solvay), N-methyl-2-pyrrolidone (NMP, 98 wt%, Aladdin), and polyethylene separator (PE, 25μm-thick, Asahi KASEI). 1 M lithium bis(trifluoromethanesulfonyl)imide (LiTFSI) in DME-DOL mixed solvents (DME: 1, 2-dimethoxy-ethane; DOL: 1, 3-dioxolane, volume ratio of 1:1) with 1wt% $LiNO_3$ obtained from Duoduo was used as the electrolyte. All chemicals were used without further treatment.

### Synthesis of CuS/CNTs

2 g CNTs were dispersed into 80 mL deionized water by ultra-sonication for 30 minutes. Then, 1g PVP and 0.5 mM $CuSO_4$ were added and ultrasound for another 30 minutes to obtain liquid A. Liquid B contained 30 mL deionized water, 0.5 mM $Na_2S$, and 1g PVP was dropwised into liquid A with strong stirring. After stirring for another 30 minutes, the obtained CuS/CNTs composite was washed several times with deionized water and ethanol and dried under vacuum at 60°C overnight.

### Fabrication of Separator

CuS/CNTs were mixed with PVDF in a mass ratio of 9:1 in NMP to form homogeneous slurry, which was coated onto one side of the commercial PE separator by blade casting. The obtained PE membrane with CuS/CNTs interlayer was vacuum-dried at 60°C for 24 hours and cut into discs with diameter of 19 mm. The raw CNTs-coated membrane was fabricated by the same procedure.

### Preparation of $Li_2S_6$ Solutions

0.05 M $Li_2S_6$ solution was prepared by dissolving 46 mg $Li_2S$ and 155 mg sublimed S in a molar ratio of 1:5 in 20 mL tetrahydrofuran (THF). The mixture was

stirred at 60°C for 24 hours to obtain $Li_2S_6$ solution.

**Static adsorption calculation of CuS/CNTs to $Li_2S_6$**

In order to clarify the polysulfide block of as-prepared CuS/CNTs, CuS/CNTs with different amounts (10, 20, 30, and 35 mg) were added to 5 mL $Li_2S_6$ solution (10 mM, corresponding $Li_2S_6$ content of 50 $\times 10^{-3}$ mmol). After 2 hours, 30 mg CuS/CNTs can effectively block $Li_2S_6$ with transparent solution. The corresponding UV-vis shows that the additions of 30 and 35 mg CuS/CNTs have similar absorbance curves. We can speculate that 30 mg CuS/CNTs could achieve limited $Li_2S_6$ adsorption. Accordingly, the adsorption of CuS/CNTs to $Li_2S_6$ is approximate $1.67 \times 10^{-3}$ mmol ($Li_2S_6$)/mg (CuS/CNTs).

**Adsorption calculation of CuS/CNTs to $Li_2S_6$ in realistic Li-S battery system**

In Li-S battery system, the mass loading of CuS/CNTs is ~0.3 mg $cm^{-2}$ ($\Phi$ = 1.9 cm), corresponding to $1.42 \times 10^{-3}$ mmol $Li_2S_6$ maximum adsorption.

$$0.3 \text{ mg cm}^{-2} \times 3.14 \times (1.9/2 \text{ cm})^2 \times 1.67 \times 10^{-3} \text{ mmol } (Li_2S_6)/\text{mg (CuS/CNTs)}$$
$$\approx 1.42 \times 10^{-3} \text{ mmol}$$

With S loading of 1 mg $cm^{-2}$ ($\Phi$ = 1.2 cm), the transformed $Li_2S_6$ is ~$5.9 \times 10^{-3}$ mmol during the discharge process (S $\rightarrow$ 1/6 $Li_2S_6$), which is more than three times to that of maximum adsorption, let alone in the case of 4.5 mg $cm^{-2}$.

$$1 \text{ mg cm}^{-2} \times 3.14 \times (1.2/2 \text{ cm})^2 / 32 \text{ g mol}^{-1}/6$$

$$\approx 5.9 \times 10^{-3} \text{ mmol}$$

**Morphological and Structural Characterizations**

The morphology of all the samples was investigated using a field-emission scanning electron microscope (SEM, Zeiss Merlin Compact) operated at an accelerating voltage of 5 kV. X-ray diffraction (XRD) patterns of all the samples were collected on an X'Pert PRO MPD diffractometer equipped with Cu K$\alpha$ radiation. Raman spectra were collected by using a LABRAM-HR confocal laser micro-Raman

spectrometer. X-ray photoelectron spectroscopic (XPS) spectra were collected on an ESCALAB250Xi X-ray photoelectron spectrometer equipped with a monochromatic X-ray source. Thermogravimetic analysis was performed in a $N_2$ atmosphere on a thermal analyzer (STA449F3) with a heating rate of 10 K $min^{-1}$. Atomic force microscopic (AFM) images were collected on a Veeco DI Nanoscope MultiMode V system. $N_2$ sorption isotherms were conducted on a Quantachrome Autosorb-IQ. The degassing of bare carbon material was carried out at 120 °C, and for the S/C composite material, the process was performed at room temperature to avoid any sublimation of S into the instrument. A long degas duration of 12 hours was applied to ensure a complete removal of air from the sample.

**Electrochemical Characterization**

The as-synthesized S/C composite was mixed with PVDF dissolved in NMP at a weight ratio of 9:1 to form a uniform slurry. The slurry was then pasted on an Al foil, dried under normal pressure at 60 °C overnight, and cut into circular discs with a diameter of 12 mm. The S loading is about 1 mg $cm^{-2}$ and the high S loading is 4.5 mg $cm^{-2}$. Coin type (CR2025) LSBs were assembled in an Ar-filled glovebox (with oxygen and water contents $< 0.1$ ppm) paired with Li anodes. It should be noted that 40 μL electrolyte was added for each battery during its assembly. Galvanostatic charge-discharge (GCD) cycling tests of LSBs were carried out on LAND instrument between 1.7 and 2.8 V (vs $Li^+$/Li). The cyclic voltammetry (CV) and electrochemical impedance spectroscopy (EIS) measurements were performed on an AUTOLAB electrochemical workstation in a potential window of 1.7−2.8 V at 0.1 mV $s^{-1}$ and in a frequency range of 0.1 Hz−100 KHz at a 0.5 mV $s^{-1}$ amplitude, respectively.

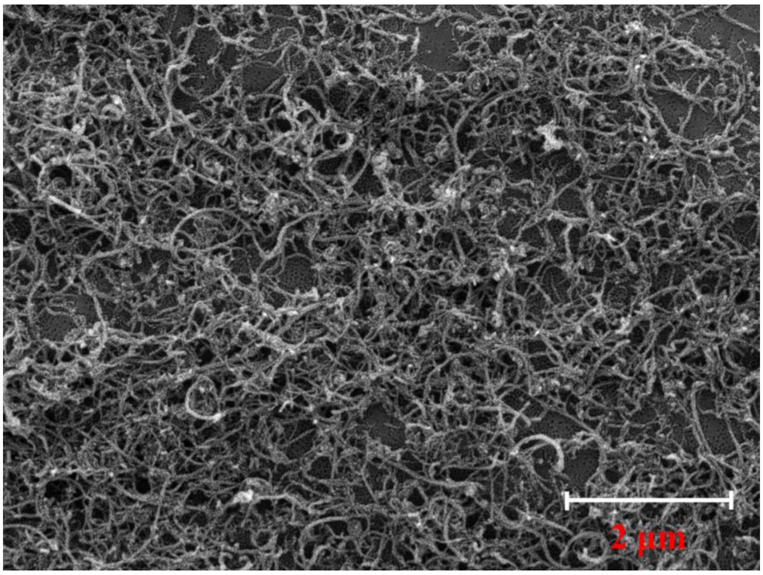


Figure S1. SEM image of CNTs.

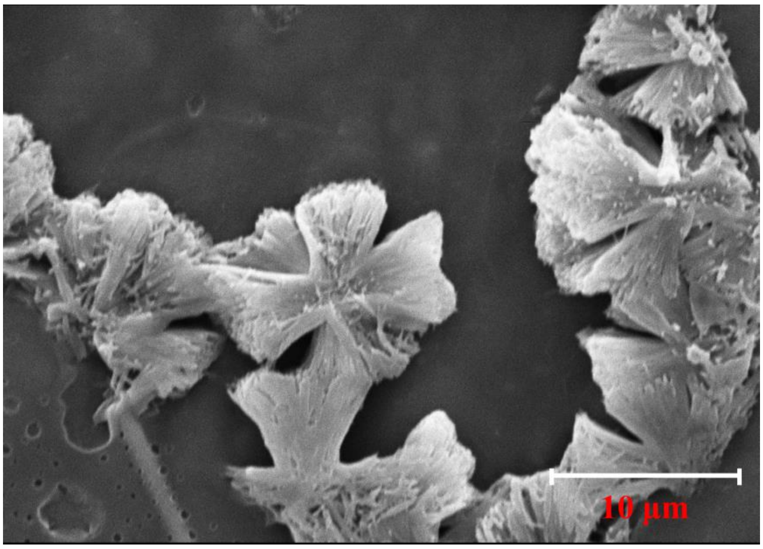


Figure S2. SEM image of CuS.

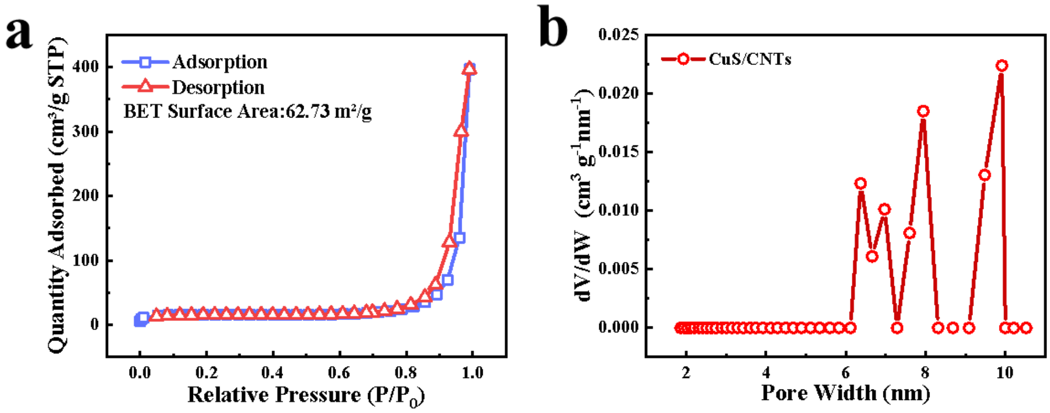


Figure S3. (a) N2 adsorption–desorption isotherms and (b) CuS/CNTs porous width.

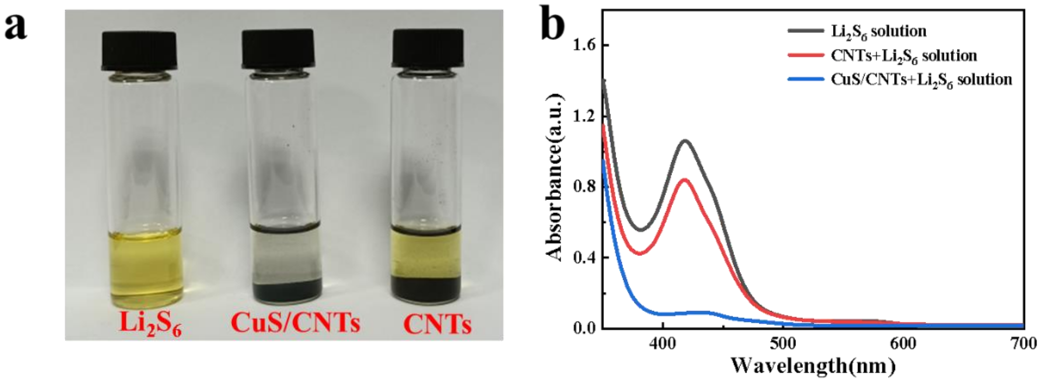


Figure S4. (a) Visual adsorption and (b) UV–visible Spectroscopy tests of $Li_2S_6$ solution upon addition of CNTs or CuS/CNTs.

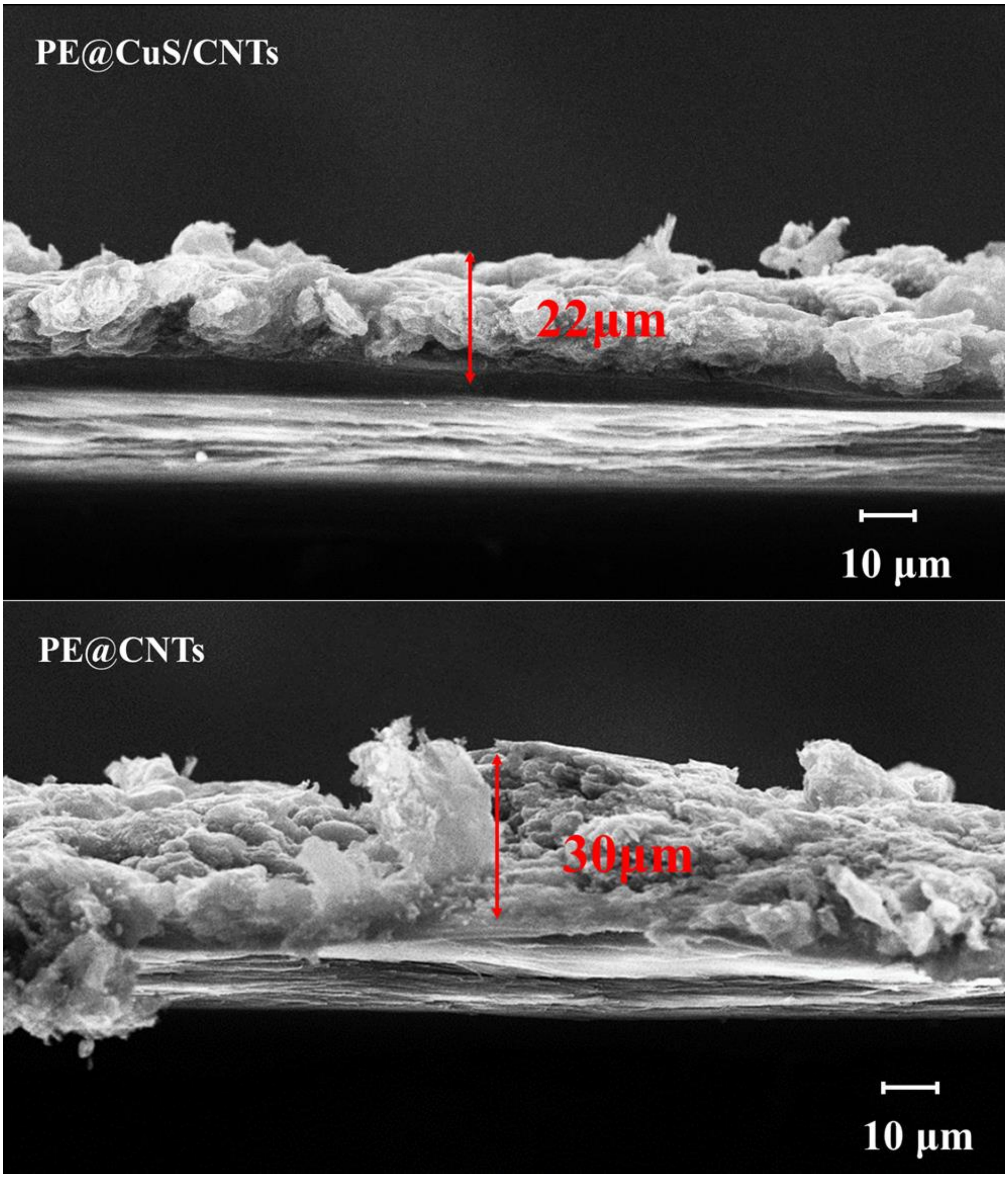


Figure S5. SEM images of the cross section of CuS/CNTs and CNTs.

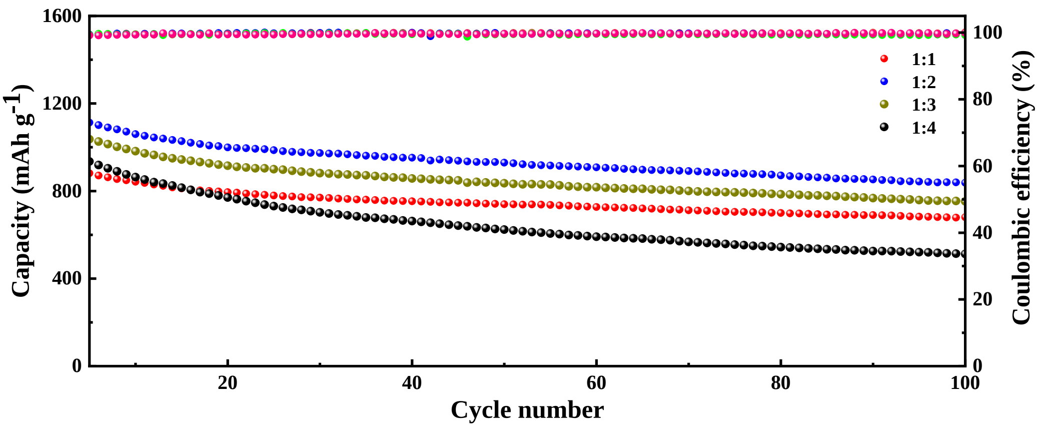


Figure S6. Cycling performances of LSBs with different CuS: CNTs ratio based on raw materials at 0.5 C.

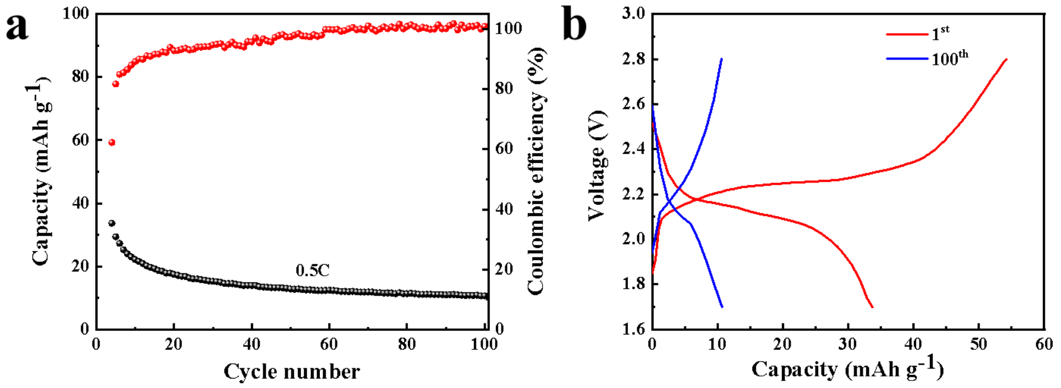


Figure S7. (a) Cycling performance of Li // CuS/CNTs batteries at 0.5C, and corresponding (b) GCD curves at the 1st and 100th cycle. (Using CuS/CNTs instead of S as the active material, the electrochemical performance is tested under the same conditions. And the CuS/CNTs loading is about 1.2 mg $cm^{-2}$.)

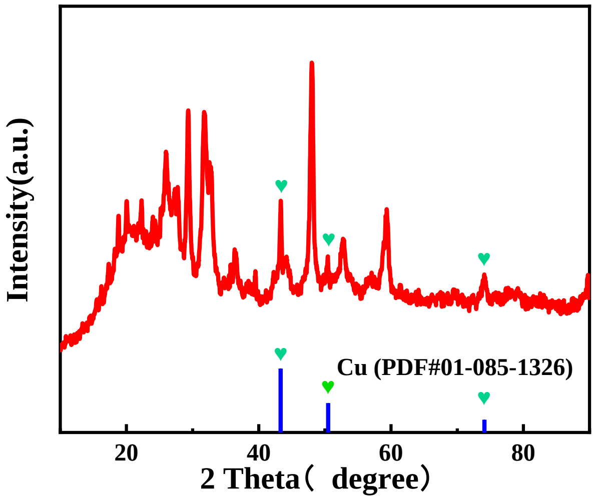


Figure S8. The XRD pattern of CuS/CNTs in Li // CuS/CNTs batteries at 0.5C after the first discharge.

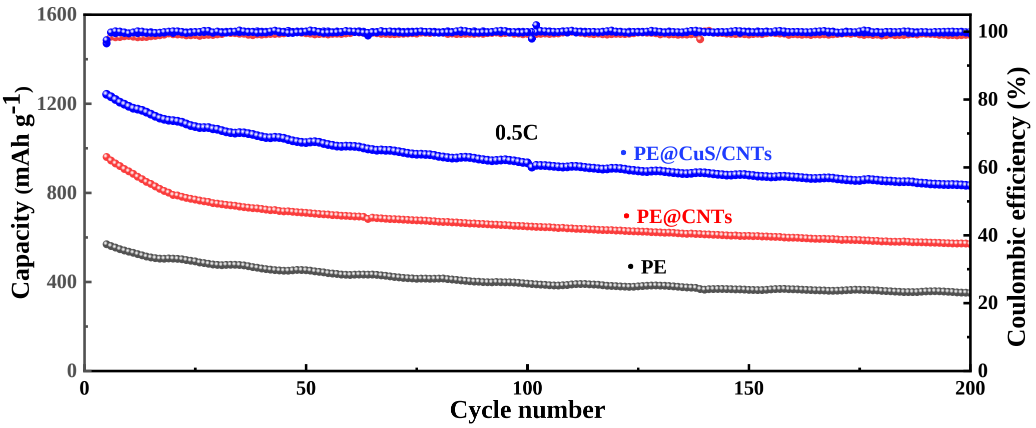


Figure S9. Cycling performance of the cells with PE separator, PE@CNTs and PE@CuS/CNTs at 0.5 C.

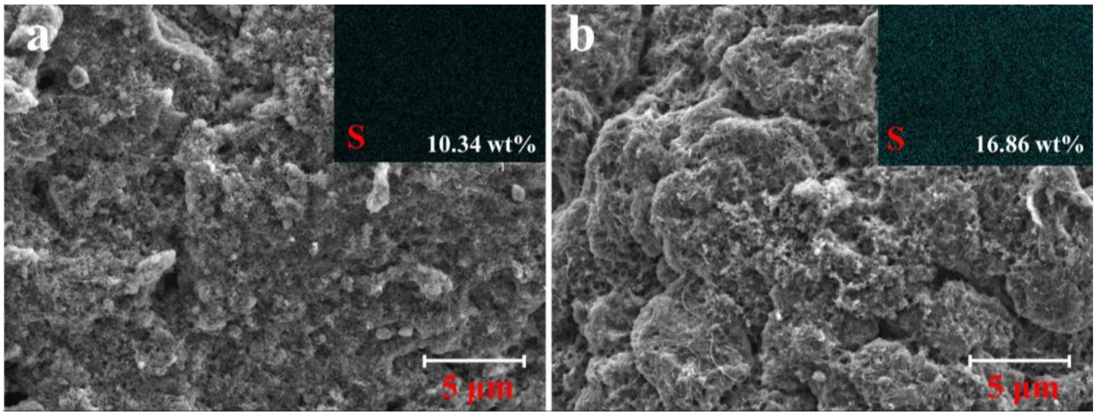


Figure S10. The top-view SEM images of (a) PE@CuS/CNTs and (b) PE@CNTs after 200 cycles.

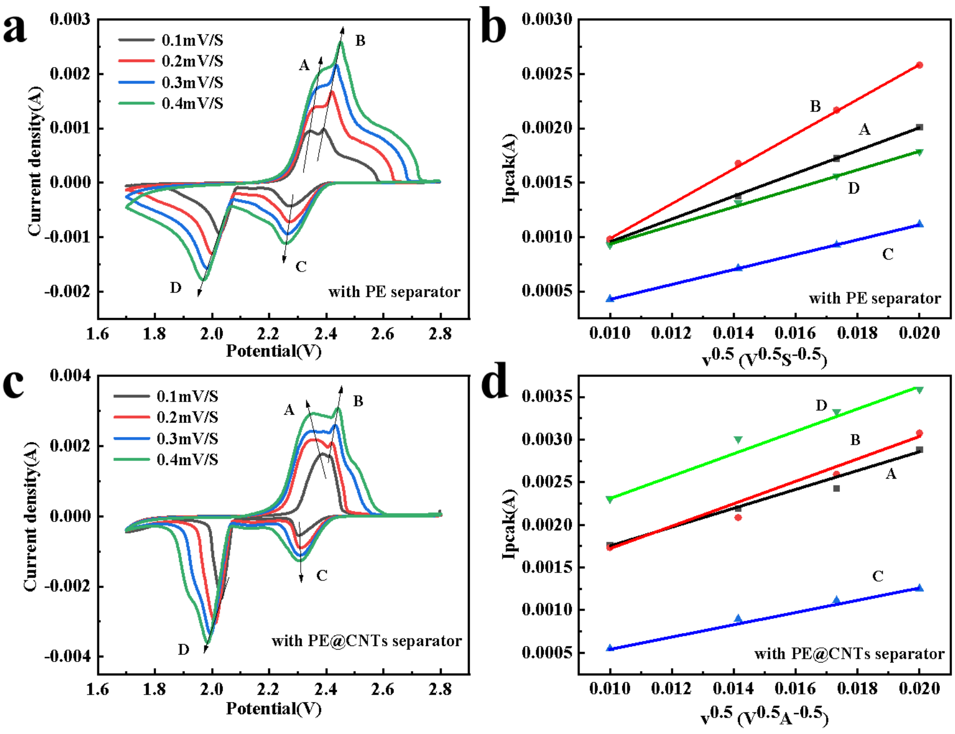


Figure S11. CV curves at various voltage scan rates and corresponding linear fits of the peak current of LSBs with (a, b) PE and (c, d) PE@CNTs.

The lithium-ion diffusion coefficients ($D_{Li+}$) were calculated from the simplified Randles–Sevcik equation:

$I_p = 2.69 \times 10^5 \times n^{1.5} \times A \times D_{Li+}^{0.5} \times C_{Li+} \times v^{0.5}$

Where Ip is the peak current, n is the number of electrons in the reaction, A is the electrode area, $C_{Li+}$ is the Li+ concentration in the electrolyte and v is the CV scan rate.

Table S1. Comparison of selected interlayers with various polymers in LSBs

| Interlayer | Cathode (S/C/PVDF) | S loading (mg cm$^{-2}$) | Electrochemical performance |
|---|---|---|---|
| This work | 6/3/1 | 1.0 | 568.5 mAh g$^{-1}$ after 1000 cycles at 2 C |
| | | 4.5 | 450 mAh g$^{-1}$ after 100 cycles at 1 C (E/S = 9.4 mL g$^{-1}$) |
| MCNT@PEG (Ref. 12) | 6/3/1 | 1.5 | 490 mAh g$^{-1}$ after 500 cycles at 0.5 C |
| MnS/CNFs (Ref. 25) | 7/2/1 | 2.0 | 579 mAh g$^{-1}$ after 400 cycles at 1 C |
| PFIL (Ref. 16) | 6.5/2.5/1 | 1.5 | 533 mAh g$^{-1}$ after 300 cycles at 2 C |
| $Mo_2C$@CNF (Ref. 32) | 6/3/1 | 1.0 | 500 mAh g$^{-1}$ after 500 cycles at 1 C |
| PCNFs-2 (Ref. 23) | 7/2/1 | 1.5 | 609.1 mAh g$^{-1}$ after 200 cycles at 0.2 C |
| | | 7.5 | 5.22 mAh cm$^{-2}$ after 100 cycles at 0.2 C |
| PPTA/CF-CA (Ref. 24) | 7/2/1 | 2.0 | 631 mAh g$^{-1}$ after 200 cycles at 0.2 C |
| CuS@GO (Ref. 39) | 8/1/1 | 1.8 | 568 mAh g$^{-1}$ after 100 cycles at 3 C |
| $TiO_2$ (Ref. 10) | 7/2/1 | 1.0 | 762 mAh g$^{-1}$ after 180 cycles at 0.1 C |
